# Diagnostic Challenges in Late Onset Multiple Acyl-CoA Dehydrogenase Deficiency: Clinical, Morphological, and Genetic Aspects

**DOI:** 10.3389/fneur.2022.815523

**Published:** 2022-03-03

**Authors:** Antonino Lupica, Rosaria Oteri, Sara Volta, Daniele Ghezzi, Selene Francesca Anna Drago, Carmelo Rodolico, Olimpia Musumeci, Antonio Toscano

**Affiliations:** ^1^Department of Biomedicine, Neuroscience and Advanced Diagnostic (BIND), University of Palermo, Palermo, Italy; ^2^Unit of Neurology and Neuromuscular Disorders, Department of Clinical and Experimental Medicine, University of Messina, Messina, Italy; ^3^Department of Neurosciences, University of Padova, Padova, Italy; ^4^Unit of Medical Genetics and Neurogenetics, Fondazione IRCCS (Istituto di Ricovero e Cura a Carattere Scientifico) Istituto Neurologico Carlo Besta, Milan, Italy; ^5^Department of Pathophysiology and Transplantation, University of Milan, Milan, Italy

**Keywords:** ETFDH, lipid storage myopathies, riboflavin, muscle biopsy, acylcarnitines

## Abstract

**Background:**

Multiple acyl-CoA dehydrogenase deficiency (MADD) is an autosomal recessive disorder of fatty acid oxidation due to deficiency of the mitochondrial electron transfer chain. The late-onset form is characterized by exercise intolerance, muscle weakness, and lipid storage in myofibers. Most MADD patients greatly benefit from riboflavin supplementation.

**Patients and methods:**

A retrospective study was conducted on patients with a diagnosis of vacuolar myopathy with lipid storage followed in our neuromuscular unit in the last 20 years. We selected 10 unrelated patients with the diagnosis of MADD according to clinical, morphological, and biochemical aspects. Clinical features, blood tests including serum acylcarnitines, EMG, and ENG were revised. Muscle biopsy was performed in all, and one individual underwent also a sural nerve biopsy. Gene sequencing of *ETFA, ETFB*, and *ETFDH* was performed as a first-tier genetic analysis followed by next-generation sequencing of an hyperCKemia gene panel in patients with undefined genotypes.

**Results:**

Clinical evaluation at onset in all our patients showed fatigue and muscle weakness; four patients showed difficulties in chewing, three patients complained of dysphagia, two patients had a dropped head, and a patient had an unexpected ataxia with numbness and dysesthesia. Laboratory blood tests revealed a variable increase in serum CK (266–6,500) and LDH levels (500–2,000). Plasma acylcarnitine profile evidenced increased levels of different chains intermediates. EMG was either normal or showed myogenic or neurogenic patterns. NCS demonstrated sensory neuropathy in two patients. Muscle biopsies showed a vacuolar myopathy with a variable increase in lipid content. Nerve biopsy evidenced an axonal degeneration with the loss of myelinated fibers. *ETFDH* genetic analysis identifies 14 pathogenic variants. Patients were treated with high doses of riboflavin (400 mg/die). All of them showed a rapid muscle strength improvement and normalization of abnormal values in laboratory tests. Neuropathic symptoms did not improve.

**Conclusion:**

Our data confirmed that clinical features in MADD patients are extremely variable in terms of disease onset and symptoms making diagnosis difficult. Laboratory investigations, such as serum acylcarnitine profile and muscle biopsy evaluation, may strongly address to a correct diagnosis. The favorable response to riboflavin supplementation strengthens the importance of an early diagnosis of these disorders among the spectrum of metabolic myopathies.

## Introduction

Multiple acyl-CoA dehydrogenation deficiency (MADD) (OMIM #231680) is a disorder of oxidative metabolism with a broad range of clinical severity (1).

The clinical phenotype has been classified according to age at onset and severity in three groups: neonatal onset with congenital anomalies, such as cystic renal dysplasia (type 1), neonatal-onset without anomalies (type 2), and late-onset (type 3) MADD (2, 3).

The latter form is the mildest, with predominant involvement in skeletal muscle (4, 5). Symptoms are highly variable and characterized by muscle involvement (myalgia and weakness), sometimes associated with recurrent episodes of lethargy, vomiting, hypoglycemia, metabolic acidosis, and hepatomegaly.

In MADD, multiple dehydrogenation reactions are impaired because of the defective transfer of electrons from a number of primary flavoprotein dehydrogenases to the mitochondrial respiratory chain. At least 12 flavoprotein dehydrogenase enzymes seem to be affected, all of which use flavin adenine dinucleotide (FAD) as the redox prosthetic group. From the FAD prosthetic group, electrons are delivered to ubiquinone in the respiratory chain *via* two other flavoproteins—electron transfer flavoprotein (ETF) and ETF: ubiquinone oxidoreductase (ETF:QO) (2). ETF is localized in the mitochondrial matrix as a heterodimer of α- and ß-subunits and contains one FAD prosthetic group and one adenosine 5-monophosphate (AMP) (6).

Electron transfer flavoprotein:ubiquinone oxidoreductase encoded by the *ETFDH* gene is a 64-kDa protein associated with the inner mitochondrial membrane. ETF-QO is composed of 11 α-helices and 19 β-strands and consists of three functional domains closely packed, sharing some structural elements: FAD-binding domain, 4Fe-4S cluster domain, and UQ-binding domain (7–9).

Many patients with MADD have variants in *ETFA, ETFB*, or *ETFDH* (2, 7–9). More than 800 MADD patients were so far reported but the screening of these three genes failed to find causative variants in some of them (10).

Interestingly, the majority of cases with adult-onset show a great response to riboflavin administration with regression of clinical symptoms and normalization of laboratory abnormalities. For this reason, this form is also called riboflavin-responsive MADD (RR-MADD).

Other genetic defects associated with this condition have more recently been described in young patients who involve riboflavin or FAD transporters and FAD synthase (11–14).

We herein report a cohort of 10 patients with MADD and a positive response to riboflavin therapy.

## Patients and Methods

A number of 10 unrelated patients (7 men) with the age at onset ranging from 12 to 62 years were diagnosed in our neuromuscular unit in the last 20 years. None of these patients reported family history of neuromuscular disorders. Patients complained about heterogeneous symptoms which include premature fatigue, exercise intolerance, muscular weakness, difficulties in chewing, dysphonia, and dysphagia. Onset was often sudden or rapidly progressive, sometimes after a trigger factor, such as gastroenteritis (one patient), statins treatment after myocardial infarction (one patient), and hypothyroidism (two of our patients). All our patients were evaluated at baseline and after 8 weeks and then with follow-up visits every 6 months for at least 2 years.

We routinely performed blood examinations which include CK levels, transaminase, LDH, and serum acylcarnitine profile. Blood screening for metabolic, hormonal dysfunctions or vitamin deficiencies was performed to exclude acquired causes of myopathy and neuropathy. A blood smear was performed to check for the presence of lipid-laden vacuolated granulocytes (Jordan Anomaly).

Nerve conduction studies (NCSs) and conventional electromyography (EMG) were performed in all our patients. A neuromuscular junction disorder was ruled out by a single fiber EMG. Muscle biopsy was performed in all patients mainly at the left quadriceps.

One patient underwent a sural nerve biopsy. Morphological studies were performed according to standard procedures. Western blot for ETFDH was performed in all our patients, using an anti-ETFDH monoclonal antibody (Abcam).

The entire coding region and intron–exon boundaries of the *ETFA, ETFB*, and *ETFDH* genes were sequenced. In a second tier, in patients with single heterozygous variants, a set of 78 genes associated with hyperCKemia or similar conditions (*ACADM, ACADVL, ACADS, CPT1A, CPT2, FLAD1, HADHA, HADHB, LPIN1, PNPLA2, SLC22A5, SLC37A4, SLC52A2, PGYM, PGYl, PGYB, PRKAG2, PHKB, PHKA1, PHKA2, PGM1, PGK1, PGAM2, PFKM, LDHA, GYS2, GYS1, GAA, G6PC, ENO3, ALDOA, AGL, ANO5, CACNA15, CAV3, CHKB, DMD, DYSF, FKRP, FKTN, RBCK1, RYR1M, SYL1, ATP5d, DGUOK, FDX1L, ISCU, MT-CO1M MT-CO2, MT-CO3, NT-CYB, POLG, ACE, ACTN3, CCL2M GAPDH, GCDH, IGF2, ILA6, LPIN2, LPIN3, MYLK, PPP1R3B, PRKAG3, TANGO2, TNF*, and *TSEN54*) were analyzed using a custom-designed panel. The sequencing covered these genes for over 99.7% of the target regions.

Patients were treated with oral riboflavin 400 mg/die except for the two young patients that were treated with 200 mg/die. Patients were followed over the years with follow-up ranging from 2 to 15 years.

## Results

### Clinical Results

At the onset, all our patients complained of myalgia and gait disturbances; these features were associated with other symptoms which include difficulties in chewing, dysphagia, dysphonia, and dropped head. Interestingly, only two patients showed an upper limb weakness where a patient manifested, a part from dysphonia and dysphagia, also a dropped head in the context of a more generalized muscle weakness.

Two patients showed ataxic gait numbness and paresthesia at lower limbs, which suggests a peripheral nervous system involvement. Both patients were screened for all acquired causes of neuropathy without any findings. Metabolic or nutritional deficiency causes of neuropathy were ruled out as well as infective of parainfective causes. None declare the use of medications potentially impairing fatty acid beta-oxidation (e.g., ranolazine).

Clinical findings at onset are summarized in Table 1.

**Table 1:** Clinical, biochemical, and morphological features in 10 patients with MADD.

**N**.	**Age at onset (years)**	**Main clinical features**							**Other symptoms**	**Trigger factor**	**CK (UI/L) at diagnosis**	**Plasma acylcarnitine profile**	**Muscle biopsy**
		**Myalgia**	**Exercise intolerance**	**Muscle weakness**	**Dysphonia**	**Dysphagia**	**Contractures/cramps**	**Chewing difficulties**					
1	29	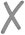	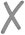	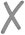	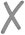	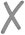		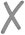	Eyelid ptosis, weight loss, Dropped head	Unknown	980	Increase of C5–C18	LSM
2	12	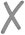	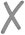	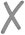						Unknown	266	Increase of C4–C18	LSM
3	42		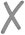	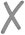				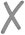	Dropped head	Unknown	1,500	Increase of C8–C18	LSM
4	40	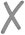		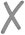					Weight loss	Unknown	500	Increase of C6–C18	LSM
5	15		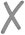	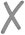			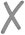			Unknown	684	Increase of C8–C18	LSM
6	47			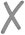				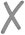		Hypothyroidism	889	Increase of C5–C18	LSM
7	62	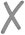				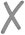			Numbness ataxia	Statins	2,250	Increase of C5–C18	LSM
8	50		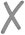	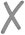			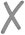	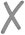	Paresthesia, ataxic gait	Gastroenteritis	368	Increase of C5–C18	LSM
9	53		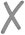	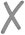		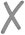	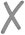			Heart attack, Statins	6,500	Increase of C5–C18	LSM
10	45	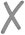		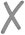						Hypothyroidism	2,000	Increase of C4–C18	LSM

*LSM, Lipid storage myopathy*.

### Laboratory Results

Serum CK levels were variably increased ranging from 260 to 6,500 in all patients. LDH available for 8 pts was 3 to 6 times normal values. Blood smear examination was normal in all patients.

Serum acylcarnitine profile, at the time of diagnosis, revealed increased medium- and long-chain acylcarnitine species (from C5 to C18).

### Neurophysiological Results

Nerve conduction studies performed in all our patients were normal except for the two patients with neuropathic symptoms in whom revealed an axonal mainly sensory neuropathy (pt. 7 and 8).

Needle EMG was normal in six out of 10 patients; five patients had a myopathic pattern with motor unit potentials (MUPs) that reduced in amplitude and duration; increased spontaneous activities with positive sharp waves (PSWs), fibrillations, and increased polyphasic motor unit potentials were detected in two additional cases. Motor unit firing revealed an early recruitment in two cases.

### Muscle Biopsy

Hematoxylin–eosin (H&E) stain showed increased fiber size variability with the presence of some atrophic fibers; multiple sarcoplasmic small vacuoles were present, mainly in type 1 fibers.

**Figure 1:**
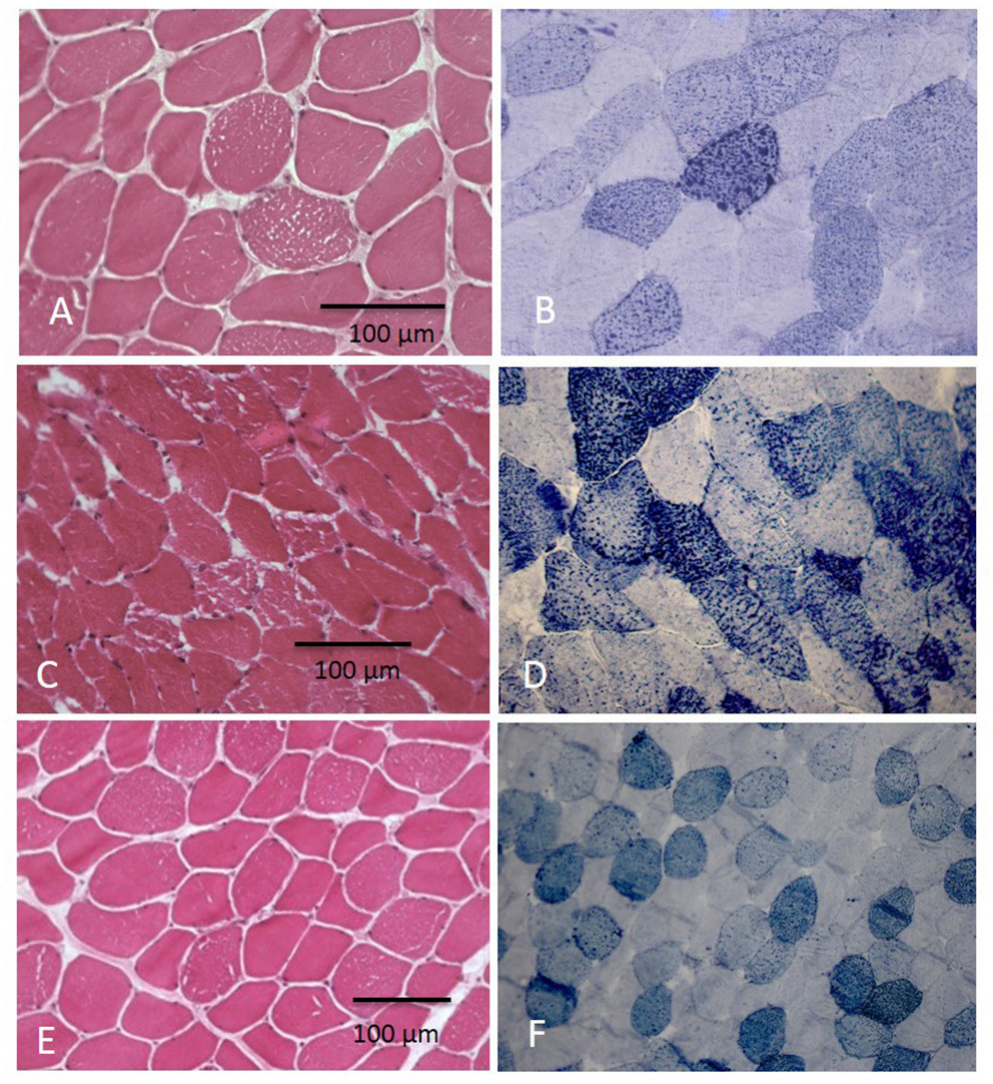
Muscle biopsy showed vacuolization in myofibers at H&E with increase of lipid content at Sudan Black at different grades: in few fibers in pt. 5 **(A,B)** and in several fibers in pts 5 and 6 **(C–F)**.

Sudan stain revealed a variable increase of lipids in muscle fibers and in particular into vacuoles (Figure 1).

**Figure 2:**
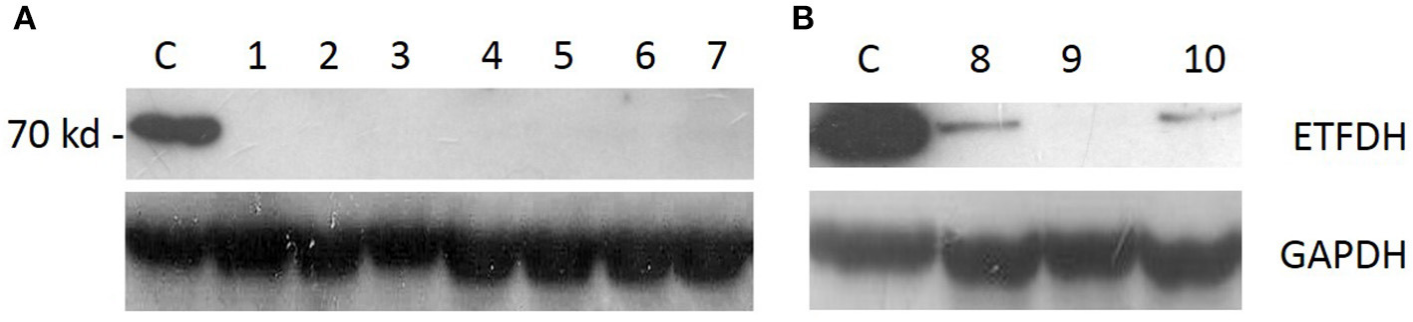
Western blot analysis: ETFDH protein expression was absent in patients with two variants [**(A)**, pts 1–7] and severely reduced in patients who harbored a single heterozygous variant [**(B)**, pts 8–10].

Western blot for ETFDH showed the absence of protein in eight patients and a marked reduction in two patients (Figure 2).

### Nerve Biopsy

**Figure 3:**
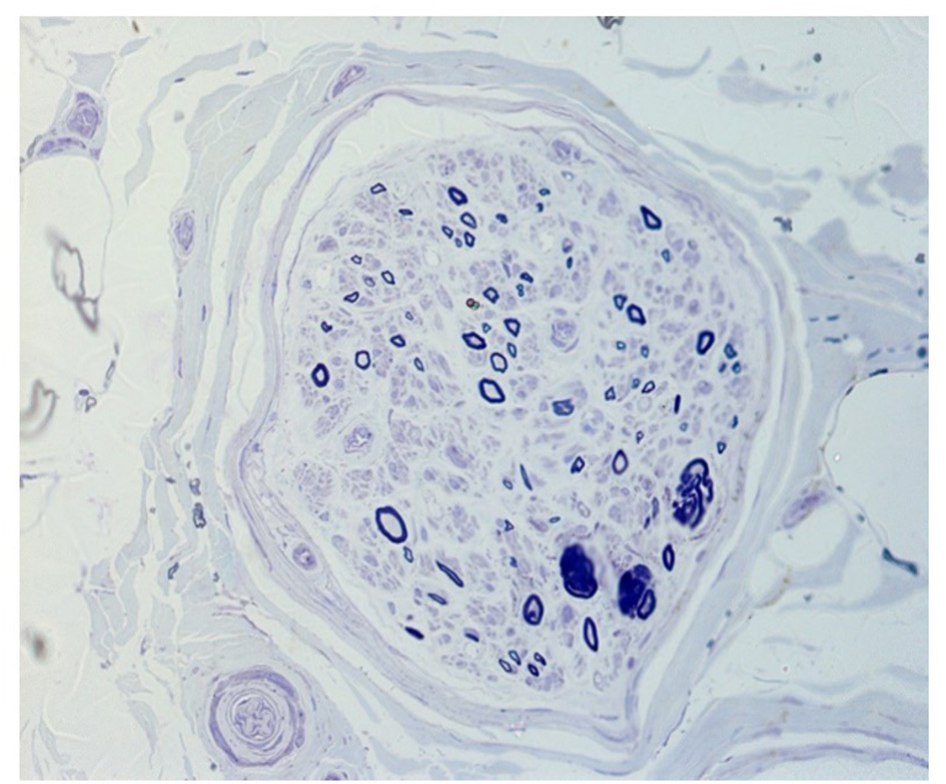
Transverse semithin section stained with toluidine blue of fascicule from a sural nerve biopsy (pt. 7) showing a severely reduced density of myelinated fibers and axonal degeneration.

Sural nerve biopsy was performed in one of the two patients with neuropathic symptoms (pt. 7) which showed a severely reduced density of myelinated fibers and axonal degeneration at transverse semithin sections stained with toluidine blue (Figure 3).

### Genetic Results

*ETFDH* gene direct sequencing was able to genetically define seven out 10 patients; two patients resulted homozygous where five patients harbored two compound heterozygous variants. Three patients carried only a heterozygous change (Table 2).

**Table 2:** Summary of the *ETFDH* gene variants identified in 10 patients.

**N**.	**Variants in *ETFDH gene* (NM_004453.4)**	**Protein (NP_004444.2)**
1	c.521T>C / c.1773_1774delAT	p.V174A/p.T591fsX2
2	c.2184T>C /c.1331T>C	p.^*^618Q/ p.V444A
3	c.1531G>A/ c.1531G>A	p.D511N / p.D511N
4	c.606+4 insT/c.1004G>C	-/ p.S335T
5	c.1366C>T/c.1828G>A	p.P456S/ p.G610R
6	c.523C>T/c.523C>T	p.R175C/ p.R175C
7	c.560C>T/c.1027T>C	p.A187V/p.W343R
8	c.1448C>T /-	p.P483L/-
9	c.293T>A/-	p.I98N/-
10	c.606+4 insT /-	-/-

* *Codon Termination*.

A total of 14 *ETFDH* different variants were identified in our cohort, and five of them were not previously reported (15). Various variants were as follows: 10 missense variants, one intronic variant, and a 2-bp deletion.

**Figure 4:**
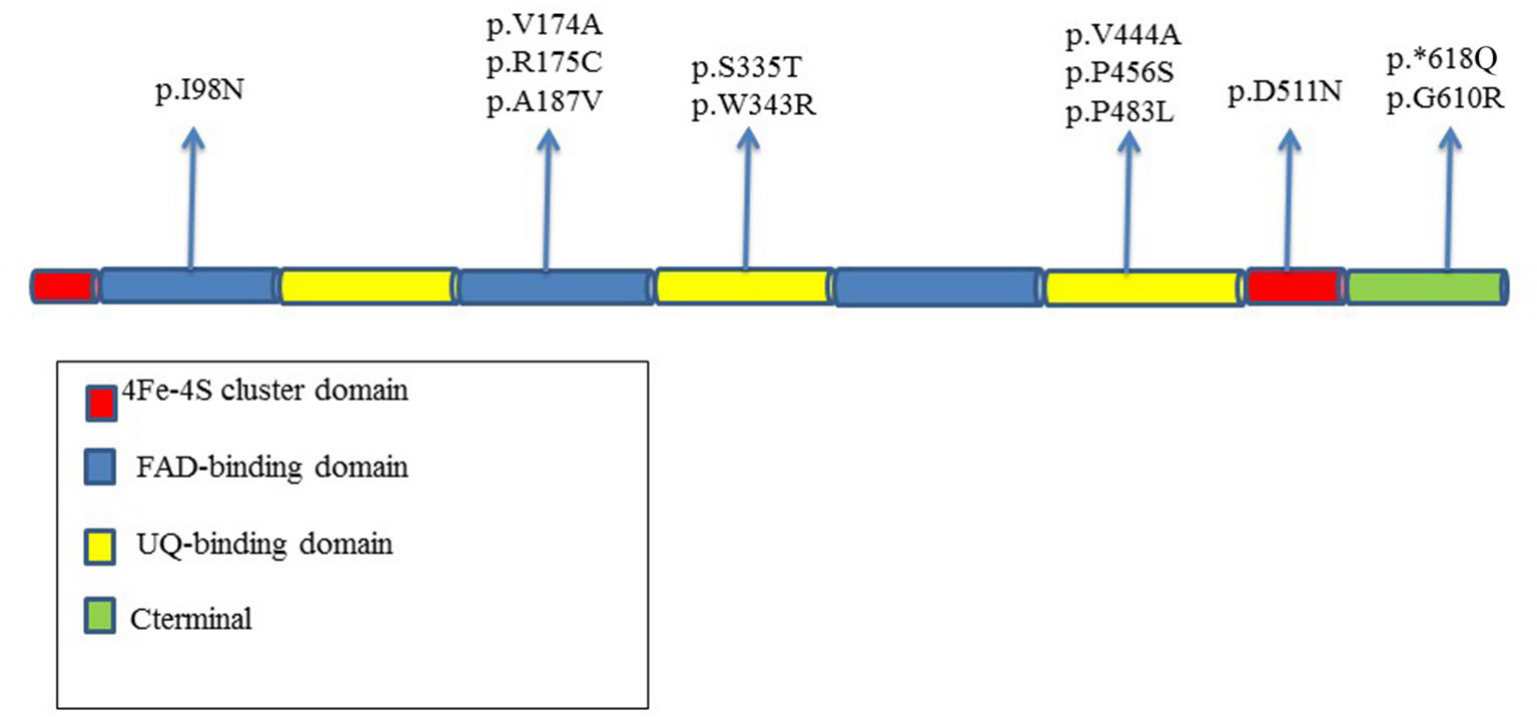
Distribution in the different domains of the protein of *ETFDH* variants reported in our MADD patients.

The distribution of the genetic changes was in the entire coding region of the gene with four variants localized in FAD-binding domain, four in UQ-binding domain, and three in C-terminal part of the protein; in a single case, variant involves the 4Fe4S cluster domain (Figure 4).

The D511N in exon 12 is located in catalytic ubiquinone region of the protein.

The P456S variant is localized in the FAD region in a moderate conserved domain, whereas G610R hits a high conserved amino acid residue in the 4Fe4S cluster domain.

Targeted next-generation sequencing for a panel of hyperCKemia-related genes did not reveal additional variants.

### Response to Therapy

All patients dramatically improved after riboflavin administration except for two cases that partially benefit of it because muscle weakness disappeared but ataxia with numbness and paresthesia persisted.

After 8 weeks from the beginning of riboflavin therapy, all our patients improved the initial symptoms and CK levels decreased of at least 50%.

After 6 months, all muscular involvement symptoms disappeared and routinely blood examination normalized in all our patients and remained stable at 2 years of follow-up.

## Discussion

MADD is a rare metabolic disorder that shares some clinical aspects with other neuromuscular disorders, which determines a very challenging diagnosis; nevertheless, an accurate diagnostic workup can allow to identify these patients to timely start the proper treatment (16).

In our experience, onset can be extremely variable and include some uncommon features, such as ataxia or paresthesia that have to be taken into account in the differential diagnosis with other muscle disorders. Another aspect to be aware when evaluating patients with a suspect of MADD is the possibility of trigger factors that can induce metabolic decompensation. In our cohort, a trigger factor was found for five patients. The identification of a stressor agent that can trigger a MADD can be very difficult. For instance, according to our findings, hypothyroidism can unmask the disease but could be a confusing factor because the condition itself may be responsible of symptoms, such as asthenia and exercise intolerance. Vomiting can be a sign of acidosis, a condition that appears in case of different metabolic disorders but can be a manifestation of MADD as occurred in some of our patients.

In MADD, laboratory findings have an essential diagnostic role. Serum CK is increased with a variable degree in all our patients, without correlation with the severity of muscle involvement or muscular pathology. Nevertheless, at follow-up, we observed a CK normalization concomitant with patients' clinical remission; this suggests its possible role as a biomarker in monitoring the clinical evolution of patients after having started a riboflavin treatment. Conversely, LDH in our patients was much more higher than CK, and this is an uncommon finding in other muscular disorders and could address clinicians to suspect the diagnosis of MADD.

All our patients were identified by serum acylcarnitine profile, confirming that this investigation is a non-invasive, fast, and reliable test for the diagnosis of MADD.

Electromyography may show a myogenic pattern but it is not specific apart from supporting a myopathic process. The presence of fibrillations or PSW, related to fiber splitting and branching phenomena, is common also in other myopathic disorders, such as inflammatory myopathies, which sometimes can be misdiagnosed in patients with MADD. The association of sensory neuropathy, even if rarely described, can be helpful in the diagnostic process. A neuropathic involvement was described in a group of Chinese patients with MADD (17); in our population, two patients showed clinical symptoms of neuropathy confirmed by NCSs. Peripheral neuropathy has been described in other few metabolic myopathies due to the alteration of lipid metabolism, such as the mitochondrial trifunctional protein deficiency (MTPD) and long-chain 3-hydroxyacyl-CoA dehydrogenase deficiency (LCHADD), as well as in Pompe disease due to an impairment of glycogen metabolism (18, 19).

In our cohort, one patient underwent a nerve biopsy that confirmed an axonal loss of myelinated fibers (Figure 3). The underlying mechanism of peripheral nerve involvement in some MADD patients is unclear, and a possible explanation is that an aberrant lipid metabolism, such as accumulation of acylcarnitine lipid intermediates and reduction of myelin lipid components in Schwann cells can induce demyelination and axonal degeneration. Some studies on the zebrafish model of MADD showed dysregulation of neurogenesis with increased neural cell proliferation, reduced motor axon branching, and hypomyelination but the exacted pathomechanism has not been detailed (20).

In our patients, muscle biopsy was quite informative, which shows invariably a vacuolar lipid storage myopathy, which suggests its crucial role in the diagnosis. Although it is an invasive procedure, and nowadays, it is considered not essential in the genetic era, in our opinion, it will be very helpful in these conditions. First of all, it can be useful to rule out other acquired causes of myopathy, and furthermore, it can address genetic analysis that sometimes quite delayed the diagnosis, especially when performed in the context of screening of gene panel.

Genetic analysis identified 14 variants and some of them were already reported in the literature. Although it is well-known that the phenotypegenotype correlation is not often clear, we compare the phenotype of our patients with the previously reported cases, and according to the same phenotype, we found that the A187V has been already described in elderly carried with a quite benign disease course that is confirmed also in our patient with a disease onset in her sixties (pt. 6) (21). On the other end, the V444R seems to be associated with a childhood onset (pt. 5) (22).

*ETFDH* genetic analysis may identify pathogenic variants but several reports in the literature describe MADD patients carrying only a single heterozygous variant or even with no evidence of variants in either *ETFDH, ETFA*, and *ETFB* genes but with a suggestive MADD diagnosis supported by the clinical history, acylcarnitine profile, and response to therapy. In our cohort, genetic analysis was able to define seven out of 10 patients; in fact, three patients carried just a single heterozygous variant. As in other reports, a possibility of cryptic variations, such as deep intronic, CNV, rearrangements, or mutation, in promoter region affects multiple exons which cannot be identified by genomic sequencing. Otherwise, it is possible that defects in another gene involved in fatty acid metabolism, or respiratory chains may act in a synergistic manner with a single ETFDH allele variant leading to an impairment of fatty acid oxidation in muscle. Finally, it is possible to assume that acquired conditions or external factors should be considered as trigger factors mainly in patients with a late onset of the disease.

In patients with a single variant, the diagnosis was supported by clinical features, acylcarnitine intermediates pattern, morphological findings, and, moreover, the response to therapy.

Response can be considered as a supportive proof for the diagnosis; however, lack of response is not conclusive because some patients have been reported as not responsive to riboflavin (15).

In our population, a prompt recovery was noted and no side effects related to riboflavin administration were observed. Muscular symptoms and laboratory abnormalities rapidly normalized whereas the neuropathy in our two patients remained unchanged; these data are consistent with previous reports on MADD associated with neuropathy that remarked that peripheral neuropathy-related symptoms are refractory to riboflavin treatment (23).

## Conclusion

Our data confirmed that clinical features in MADD patients are quite variable in terms of disease onset and symptoms making still challenging the diagnosis. Laboratory investigations as serum acylcarnitine profile and muscle biopsy may strongly address the diagnosis.

An early recognition of these disorders can allow starting a proper treatment that might result in a complete clinical recovery.

## Data Availability Statement

The datasets presented in this article are not readily available because of Ethical and Privacy restrictions. Requests to access the datasets should be directed to omusumeci@unime.it.

## Ethics Statement

The studies involving human participants were reviewed and approved by Ethic Committee AOU G. Martino di Messina, Italy. The patients/participants provided their written informed consent to participate in this study.

## Author Contributions

AL, OM, RO, and AT participated in the design of the study. AL, RO, SV, CR, and OM collected all clinical data and performed neurological examinations. OM, RO, SV, and DG provided all of the biochemical and molecular data. CR did the histopathological investigations. All authors helped in drafting the manuscript and read and approved the final manuscript.

## Conflict of Interest

The authors declare that the research was conducted in the absence of any commercial or financial relationships that could be construed as a potential conflict of interest. The reviewer CA declared a shared affiliation, with no collaboration, with one of the authors, SV, to the handling editor at the time of the review.

## Publisher's Note

All claims expressed in this article are solely those of the authors and do not necessarily represent those of their affiliated organizations, or those of the publisher, the editors and the reviewers. Any product that may be evaluated in this article, or claim that may be made by its manufacturer, is not guaranteed or endorsed by the publisher.
